# Macromolecule Particle Picking and Segmentation of a KLH Database by Unsupervised Cryo-EM Image Processing

**DOI:** 10.3390/biom9120809

**Published:** 2019-11-30

**Authors:** Miguel Carrasco, Patricio Toledo, Nicole D. Tischler

**Affiliations:** 1 Facultad de Ingeniería y Ciencias, Universidad Adolfo Ibañez, Av. Diagonal Las Torres 2700, Santiago 7941169, Chile; patricio.toledo@uai.cl; 2 Laboratorio de Virología Molecular, Fundación Ciencia & Vida, Av. Zañartu 1482, Santiago 7780272, Chile; ntischler@cienciavida.org; 3 Facultad de Medicina y Ciencia, Universidad San Sebastián, Lota 2465, Santiago 7510157, Chile

**Keywords:** cryo-EM, automatic particle selection, single particle picking

## Abstract

Segmentation is one of the most important stages in the 3D reconstruction of macromolecule structures in cryo-electron microscopy. Due to the variability of macromolecules and the low signal-to-noise ratio of the structures present, there is no generally satisfactory solution to this process. This work proposes a new unsupervised particle picking and segmentation algorithm based on the composition of two well-known image filters: Anisotropic (Perona–Malik) diffusion and non-negative matrix factorization. This study focused on keyhole limpet hemocyanin (KLH) macromolecules which offer both a top view and a side view. Our proposal was able to detect both types of views and separate them automatically. In our experiments, we used 30 images from the KLH dataset of 680 positive classified regions. The true positive rate was 95.1% for top views and 77.8% for side views. The false negative rate was 14.3%. Although the false positive rate was high at 21.8%, it can be lowered with a supervised classification technique.

## 1. Introduction

Macromolecules consisting of proteins and nucleic acid play a crucial role in all living systems, and information on their structures is essential for achieving detailed mechanistic insights into their function. Atomic level high-resolution structures can reveal antigenic surfaces and molecular interaction sites such as those involved in multimerization and binding to substrates or other molecules. Structure determination by cryo-electron microscopy (cryo-EM) linked to 3D image reconstruction has reached near-atomic resolution thanks to Bayesian image processing algorithms and recent technological advances such as direct electron detectors [1,2,3,4]. High-resolution structure determination by cryo-EM demands the processing of thousands of single-particle images and, therefore, picking single particles from electron micrographs is still considered a difficult problem and most of the time is performed manually [1,5,6].

Manual picking is a laborious and time-consuming task prone to errors, while fully automatic particle selection is far from being settled due to the numerous difficulties. One of the challenges is that micro-graphs present a low signal-to-noise ratio due to the often low-contrast of the probe and the low electron dose used to avoid destruction of the sample. Furthermore, micrographs often suffer from image distortions introduced by the microscope or detection systems and, moreover, may include heterogeneous particles which generate different 2D views in random orientations requiring classification [6,7,8].

Many particle picking algorithms have been proposed and implemented in image processing suites such as EMAN2 [9], SIGNATURE [10], DOGPICKER [11], XMIPP [12], and ARACHNID [13]. These algorithms can be organized into three categories: Template-matching, shape-recognition, and dynamical-programming. Template-matching techniques employ cross-correlation similarity in the micrographs with user pre-defined particle and noise information [14,15,16]; shape recognition algorithms identify particle information from morphological features [17,18]; finally, dynamical programming is based on a continuous machine-learning process in which the user teaches the algorithm about wrongly selected particles (false positives) [12,19,20,21,22]. Although there are trade-offs among the techniques, automatic processes like template-matching are preferred, because they avoid manual selection of a huge number of images by users. Moreover, the selection process might be hard even for trained users, mainly due to the fact of fatigue or lack of consistency.

This work presents an automated particle picking algorithm based on the composition of two image filters: Anisotropic diffusion [23] and non-negative matrix factorization (NNMF) [24]. Together, these filters allow particle detection under highly defocused conditions, mainly through shape-recognition techniques. To evaluate the performance of the proposed algorithm, we used a database of cryo-EM images for which a ground truth (picking coordinates) was available for a testing database. Below, we briefly introduce the necessary concepts associated with noise-reduction techniques and the particular characteristics of cryo-EM images before explaining in detail the method proposed and analyzing the experimental tests.

## 2. Materials and Methods

### 2.1. Preliminary

Due to the low contrast present in cryo-EM images, most of the algorithms use a background noise reduction technique. The main families described in the literature are those based on anisotropic diffusion, non-linear and adaptive filtering, general statistics, and wavelet transform [25,26,27,28,29]. Not all techniques are useful for all kinds of noise; therefore, it is important to assess the spectral properties of the image. One of the most appropriate filters for this type of image is the Perona–Malik anisotropic filter. The Perona–Malik (PM) technique allows for noise level reductions and, at the same time, border preservation from structures through anisotropic diffusion constant tuning [23,30] based on solutions of the heat equation, meaning that the diffusion constant is lower near the border and higher in uniform areas. Although cryo-EM image quality is improved, obtention of good particle–background separation performance by tuning is still difficult.

### 2.2. Image Framework and Spectral Properties 

**Figure 1:**
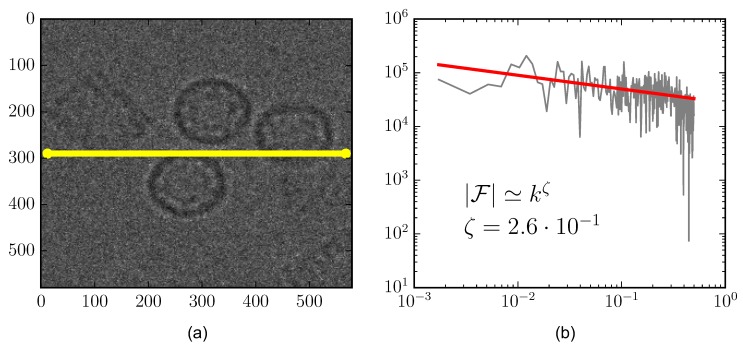
Frequency content of image. (**a**) Original image, yellow line: Level region profile; (**b**) Fourier space for yellow line profile.

Let W be an image in the space $M_{mn}\left(N\right)$ of arrays with n rows and m columns with pixels $w_{ij}$ in the natural numbers. Figure 1 shows a sampled subset of the image W at a given row (yellow line). The image on the right side shows the spectral content |F(M)| along with a power-law (pink noise) best fit line. At low and high frequencies, power-law finite-size scaling can be seen; this is a known artifact due to the image size. Pink noise is characteristic of strong correlation, scale invariance, wide dynamical range (three orders of magnitude in this case), and positivity of W; see Pruessner [31] for a thorough review. This characteristic will play a key role in the proposed segmentation strategy.

### 2.3. Matrix Decomposition NNMF

Non-negative matrix factorization (NNMF) exploits three characteristics present in cryo-EM images. The first is a local representation of the image, opposed to, for instance, a global principal component method thus attacking the problem of strong correlation in cryo-EM images. The second is the use of non-negative restrictions that only allow combinations as sums over the elements; this is a key factor in locality [24]. The third is a property related to scale-invariance. The NNMF decomposition is the result of minimizing a distance φ among an array *W* and a product *SH*, subject to the constraints:

(1) $$\{\begin{array}{cc} argmin_{SH} & \frac{1}{2}\varphi \left(W,SH\right)\\ s.t. & S_{ij},h_{ij}\geq 0 \end{array}$$

In image processing, the operator φ may be a distance or a divergence, because triangular inequality is not necessary. Examples are the Frobenius distance and the Kullback–Leibler and Itakura–Saito (IS) divergences. They show algebraic scaling which is desired when wide dynamical range is present [32]. In particular, IS divergence shows complete scale-invariance. Thus, the NNMF solution provides an approximate decomposition for image *W* in terms of:

(2) W≅SH

where the rank of S is *ξ* and H is the codification. This representation has scaling properties suitable to our analysis.

### 2.4. Proposed Algorithm

It is well known that scale-space signal separation can only be seen in areas of stable characteristics [23,33,34]. We therefore used a process similar to that proposed by Voss et al. [11]. In the special case of differences of Gaussians (DOGs), experimental results from Mikolajczyk [35] show that this is a closed approximation of a Laplacian of Gaussian; its properties are scale-invariance and information reduction with stable characteristic detection. Algorithms based on this separation are known in the literature thanks to invariant point description [34,35,36].

**Figure 2:**
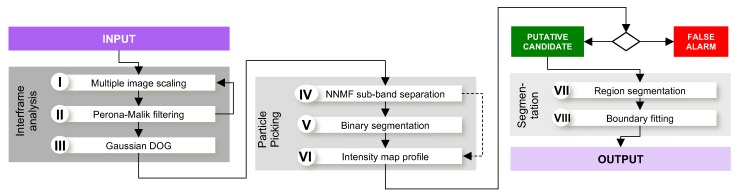
General proposed processing overview with three main phases: Interface analysis, particle picking, and segmentation (*DOG: Differences-of-Gaussians, NNMF: Non-negative Matrix Factorization*).

A single pass of a PM filter is not sufficient for stable point detection, because the signal-to-noise ratio is extremely low in cryo-EM images (less than ~10 dB). Our proposal is to decrease noise by successive application of PM filters which might be seen as a Gaussian approximation [25]. In overall terms, the proposed technique uses a mixture of scale–space filters (PM) and band separation (NNMF) for unsupervised geometric analysis of potential areas; thus, there is no prior knowledge about the desired structure inside the image. However, rightfully accepted regions are those with closed borders which we interpreted as macromolecules. To reduce false-positive cases, we implemented a border frequency algorithm (cepstrum). An overview of the process applied is shown in Figure 2. Below, we explain the theoretical definition of the model used.

### 2.5. Phase I: Interframe Analysis

**Figure 3:**
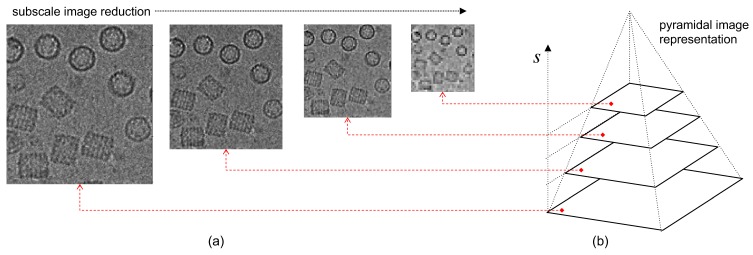
Multi-scaled Perona–Malik filtering approach: (**a**) multiple imagen level reduction, (**b**) pyramidal image representation.

Scale change is accomplished by an operator, $T_{s}$, defined over $M_{mn}\left(N\right)$ into $M_{pq}\left(N\right)$. The operator is parametrized by the scale, s (Equation (3)). In practice, we used a family of rescaled images:

(3) $${\left\{T_{s}W\right\}}_{S_{0},S_{1},\ldots ,}$$

with the scale parameter given by $s_{u}=\frac{1}{2^{u/U}}-\frac{1}{3}$ with u=0,1,…,U−1, rounded to the nearest integer as proposed by Lowe [36]. U was fixed at 16; thus, we amplified sixteen times the number of input images (see Figure 3). As discussed above, the PM filter is a solution for the diffusion equation. Details of its implementation are shown in Reference [23], and an example in the context of image regularization is shown in Reference [30].

**Figure 4:**
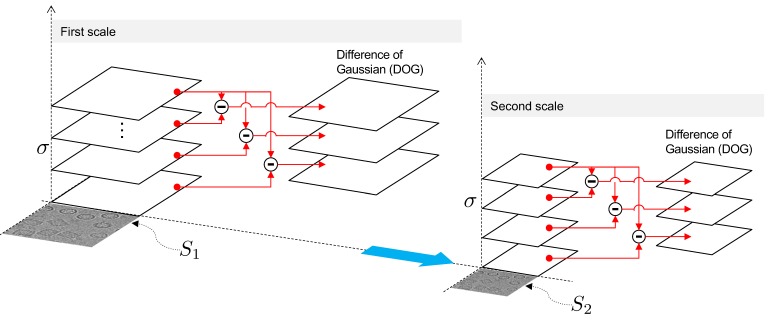
Multi-scaled Perona–Malik filtering scheme.

The PM operator depends on the diffusion which, in our case, is given by $1-exp\left(s^{2}/K^{2}\right)$ which is Gaussian in s, and by K, a regularization constant which we set at k=0.05. Graphically, this process is represented as the successive application of scale changes in the image and increments in the iterations of the PM filter as illustrated in Figure 3b. The next step is successive Gaussian application on each scale. Let us denote $G_{\sigma }$ a Gaussian kernel in $M_{mn}$ with components $g_{ij}$:

(4) $$g_{ij}=\frac{1}{2\pi \sigma^{2}}exp\left(-\frac{i^{2}+j^{2}}{2\sigma^{2}}\right)$$

with σ a standard deviation. Mathematically, this consists of applying successive convolutions, modifying the parameter σ at each scale, s, of the previous process. The difference in Gaussians (DOG) approximates the Laplace operator which may be viewed as a diffusion process [36]. Depending on the application, this operation allows for stable region detection, especially when invariant interest points are sought. However, our aim was to find regions with structures surrounding particles and not necessarily interest points; thus, we employed a robust scheme consisting of taking the difference of each image from a given octave to the last (see Figure 4).

The size of the convolutional image depends on s as described in Equation (4). To adjust all images to the same scale, we rescaled them to the original size in $M_{mn}\left(N\right)$. Then we flattened the array defined over the arrays of m rows and n columns into the vectors of dimensions mn. We note that each row of the rescaled image represents a scale–space variation from the original image W. Variations of σ per octave and s to create multiple octaves generates an array, M, with scale–space information.

### 2.6. Phase II: Particle Picking

**Figure 5:**
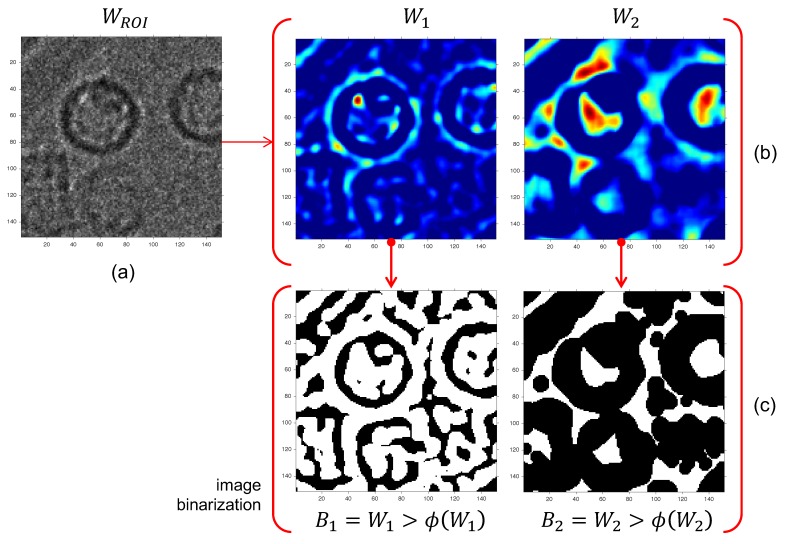
Segmentation process of W into $W_{1}$ and $W_{2}$. (**a**) Original image, (**b**) image decomposition by successive octaves (pseudo-color images), and (**c**) image binarization.

The main advantage of non-negative matrix factorization is dimension reduction through positiveness of the process. The factor controlling this reduction is ξ which is always less than mn in the case of input images W belonging to $M_{mn}\left(N\right)$. When ξ=2, the matrix S contains two columns, carrying regular structure information (particles) and background noise, respectively. Let $W_{j}$ be an array in $M_{mn}\left(N\right)$ from $s_{\cdot j}$. Figure 5 shows that particles were contained in $W_{1}$ and the background in $W_{2}$.

The region of interest (ROI) detection was conducted with a segmentation process that separates by means of a standard threshold selection algorithm. For each image $W_{i}$, an optimum threshold was selected and a binary image was produced. We used the segmentation algorithm Φ described in References [37,38] called Otsu: $B_{i}=\Phi \left(W_{i}\right),\;i=1,2,$ for its simplicity.

After the segmentation process, based on the observations of the average macromolecule size, a size range area, $A_{ROI}$, was labeled according to $100\;\mathrm{px}\leq A_{ROI}\leq 1500\;\mathrm{px}$. The size range was one of two unknown parameters used in this methodology and can be adapted by the user according to the image scale. As shown in Figure 5, background and ROIs were separated. However, as the structure shape was not known in advance, they were labeled anyway, even though most of them were false positives. Note that in the $W_{2}$ image, every ROI had a well delineated centroid. Moreover, $W_{1}$ had structure borders (open and closed). Therefore, a closed-border search technique was used as the selection method.

**Figure 6:**
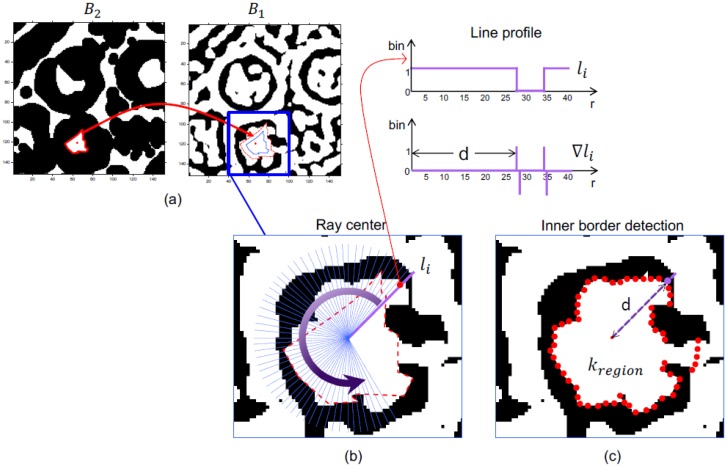
Binary image profile analysis. (**a**) B_1_ and B_2_ are binary images from the segmentation step; (**b**) the r parameter was set to 50 pixels starting from the mass center; and the (**c**) inner border detection of a region of interest.

Let K be the set which indexes the centroids, K={0,1,2,…}. Let ${\left\{c_{k}\right\}}_{k\in K}$ be the family of centroids found in $W_{2}$. For each kth centroid, a set of concentric radii ${\left\{l_{k}^{i}\right\}}_{k\in K,\;i=0,1,\ldots }$ of length r were traced, and samples of $W_{2}$ were taken over the radii. This parameter r was the second unknown parameter used for which we fixed a value of <50 px. A simple slope analysis detected the borders as shown in Figure 6a.

The derivative of the profile $l_{k}^{i}$ enabled us to detect the structure analyzed at the edge of the internal region. We note that $l_{k}^{i}$ was a binary variable along the radius. Therefore, its derivate was a series of impulses. The border d of the structure was the distance to the first positive impulse (see Figure 6b).

**Figure 7:**
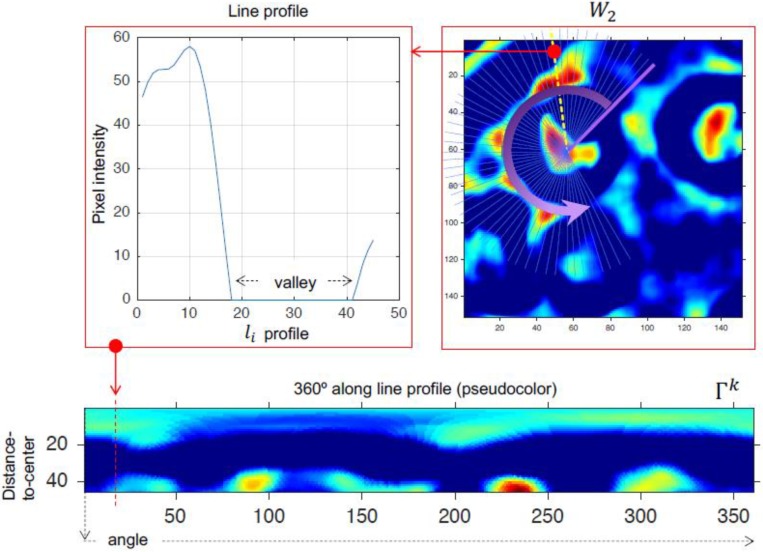
Intensity levels profile. Pseudo color: Blue = 0, red = 255.

Because of the high false alarm rate during labeling, an initial filtering process based on morphological and chromatic aspects is proposed. For every centroid, the image $W_{2}$ was sampled over the radius set, and every sampled pixel was placed side by side (Figure 6). Let $\Gamma_{k}$ be the image generated around the ROI centroid k (Figure 7). We propose an unsupervised filter by means of cepstrum analysis [39]. The cepstrum of the image generated was $C^{k}$. When the k class structure was defined as true, then region segmentation and optimal orientation were carried out.

(5) $$\{\begin{array}{cc} class_{k\in K} & C^{k}=\log\left|F\left(\Gamma^{k}\right)\right|\\ s.t. & F^{-1}\left(C^{k}\right)\;be\;pure\;real \end{array}$$

### 2.7. Phase III: Segmentation 

**Figure 8:**
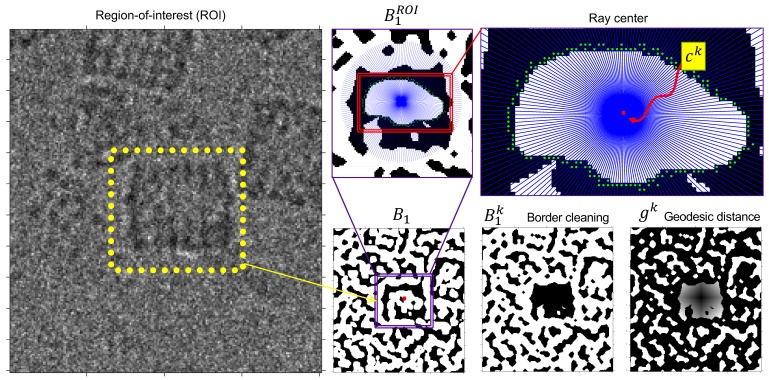
Interior region erasing and geodesic distance calculation.

The next process was proper segmentation of each structure by superposing an internal polygon on $c_{k}$. In this way, internal pixels from each region could be erased and a region with a definite border obtained (Figure 8, border cleaning). Unfortunately, cases exist where the internal borders are not closed. For this reason, we used the geodesic distance to obtain mapping of all the pixels towards the mass center of the region. To do this, let d be a geodesic function on a binary image and a start point; a distance is defined between the borders and the center of the region as: $g_{k}=d\left(B_{k},\;c_{k}\right)$.

When a region has an open border, the geodesic distance allows equal weighting to be applied within each branch of the region; this is useful because the noise present in the image means that it is not always possible to obtain a region with regularly shaped borders or else regions remain inside the structure which it has not been possible to erase.

**Figure 9:**
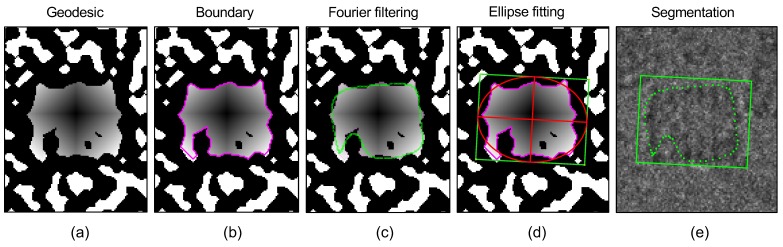
Border determination and region ellipse fitting: (**a**) geodesic distance ratio; (**b**) boundary detection; (**c**) Fourier boundary filtering; (**d**) angle estimation by ellipse fitting; (**e**) final segmentation.

Once $g_{k}$ is obtained, the coordinates of the border at a given distance were recovered. Border information contains noise; therefore, a Fourier border descriptor was used [38], maximizing the signal and minimizing noise at 98% of energy. In practice, the critical distance was set as three times the median between the region’s centroid and its border (see Figure 9, Fourier filtering)

The final step in the segmentation was the ellipse fitting over the region’s border. We used the algorithm proposed by Fitzgibbon et al. [40] which uses border coordinates and the region centroid as explained (see Figure 9, ellipse fitting). Again, noise was bypassed with an outliers search technique described in Reference [41]; thus, border points labeled as bad were rejected. Ellipse rotation angles and axis are shown in Figure 9 (segmentation).

## 3. Results

**Figure 10:**
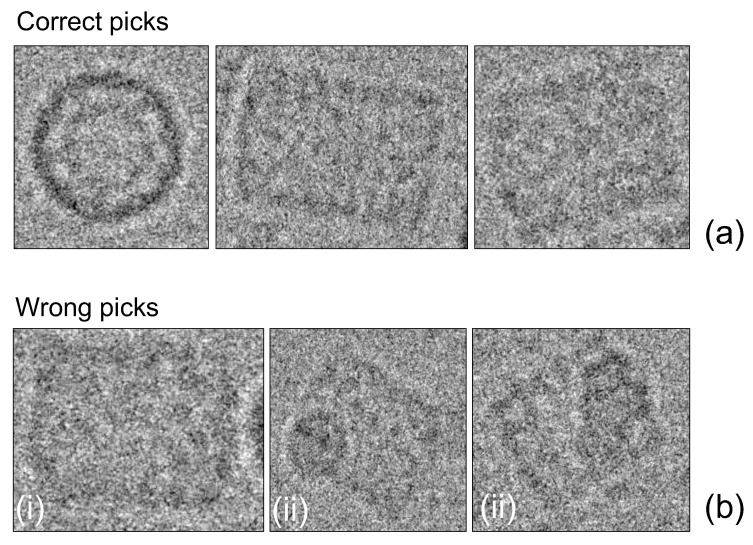
(**a**) Correct picks: No particle or any other things overlapping and side and top views must be picked. (**b**) Wrong picks: (i) no overlap with another body, (ii) two or more bodies overlapped.

Our algorithm was tested on a KLH database from the National Institute of Bioinformatics and INSTRUCT Center for Image Processing in Microscopy. The images were acquired with a Philips CM200 TEM microscope equipped with a CCD Tietz camera (4 megapixels) as focal pairs at a nominal magnification of 66,000× at 120 kV using the Leginon system [42]. The pixel size was 2.2 Å at the specimen, and the accumulated dose for each high magnification image area was 10 e-/Å^2^. No previous training on ROI was needed because of the unsupervised nature of our algorithm. Moreover, as the training dataset contains true-positive classified-region coordinates, we used them as a performance benchmark. One main difference was that the KLH dataset considers only side view particles as true positives; our analysis takes both top and side views as true positives because we used an algorithm allowing segmentation of the two regions (see details in Figure 10).

**Figure 11:**
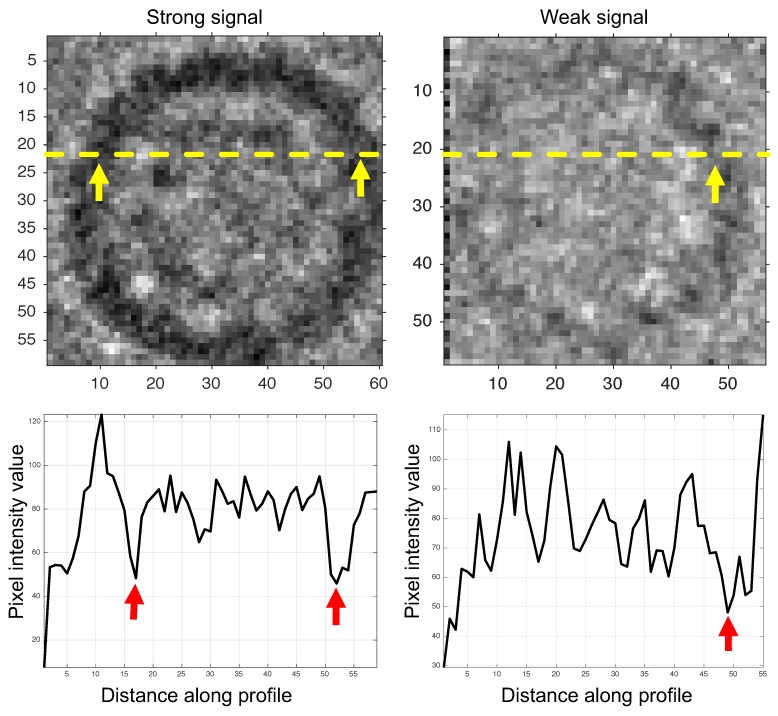
Low and high contrast level region profiles.

We checked that the training dataset had 680 true-positive classified regions by using Mouches’s criteria [43] in only 30 testing images. The particle layout was random, and the mean distance search of the algorithm was set to 45 pixels with no prior information about shape and orientation. Additionally, there was no particle segmentation limit for each micrograph. The noise level was high as can be seen in Figure 11.

**Figure 12:**
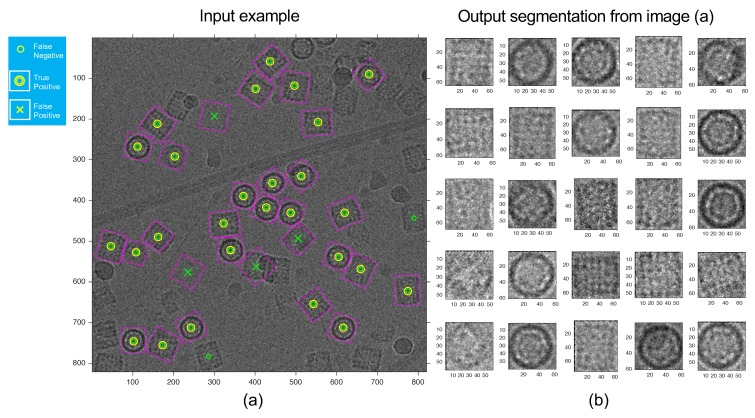
Automatic segmentation of the ROI by proposed algorithm. (**a**) Input image example, (**b**) output segmentation after an unsupervised classification process (Equation (4)) from the left image.

Most structures have low signal-to-noise ratio contrast, this implies that there is many regions with diffuse borders and a big open part. However, our algorithm can close off non-generated borders. An example of the applied segmentation is shown in Figure 12. Unlike particle search methods, our method can find a region’s structure type, enabling it to correctly re-orient a large number of regions. This allows for a more robust 3D model, because there is a previous alignment of targeted elements. Also, it is able to deal with none or multiple particles on each micrograph.

**Figure 13:**
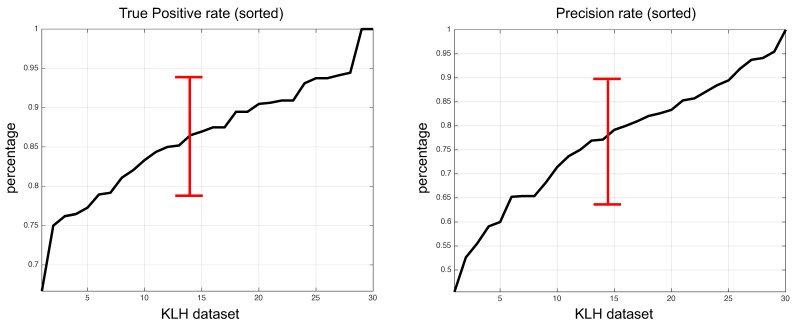
Algorithm performance over testing images of the KLH dataset. True positive rate was 86.3% ± 7.5% and the precision rate was 77 ± 13.1%. This graph shows the lowest and highest performances according to the true positive rate and the precision rate from each micrograph.

We reached a true positive rate (TPR) index or recall of 86.3% and a precision of 77.0%. By mixing the two measures, we obtained an F1-score of 81.4%. The performance curve is shown in Figure 13 for each image analyzed along with the means and standard deviations.

**Figure 14:**
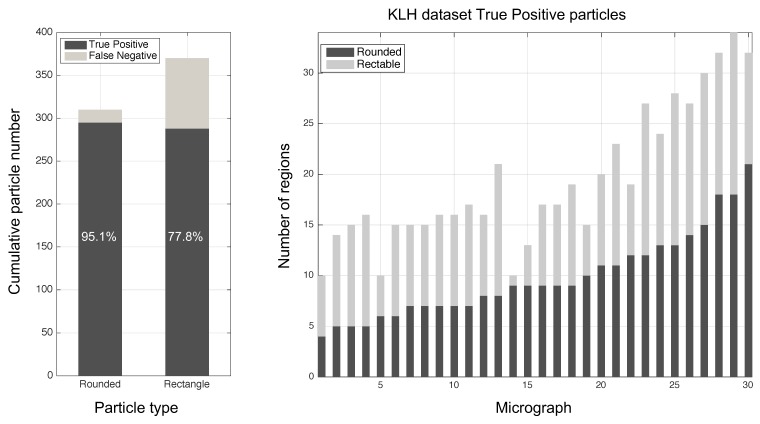
The algorithm’s performance over the testing images. Each micrograph has a different amount of particle types (rounded, rectangle). It is observed that when the number of rounded particles increase, there are more rectangle particles.

**Figure 15:**
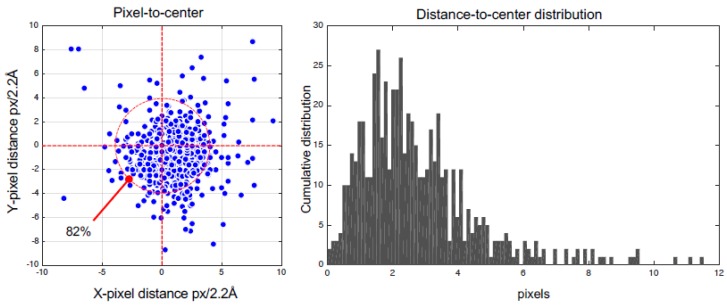
The algorithm’s performance over the testing images in the combined region.

In terms of particle class separation, 49.9% of the regions were side views and 50.1% top views (Figure 14). To assess performance, we obtained 64.6% of the particles at distances of less than 3 pixels and 82.2% at 4 pixels with respect to real centroids (Figure 15). This performance may be improved with a matching technique taking into account that there was no prior information. The centroids tested showed a mild lag of 0.862 pixels in the x-direction and −0.677 pixels in the y-direction.

## 4. Discussion

The macromolecules used in this study had both top and side views as detailed above. In general, there are three families of algorithms for structure identification: Supervised, semi-supervised, and unsupervised [44]. Table 1 presents six algorithms from the literature which operate on the same KHL database jointly with our proposal. Our proposal was able to detect both types of views and separate and align them automatically. Our top view FPR was below average, while the TPR was more than 5% higher than the average. This means that our algorithm had a high performance when the ROI presented a circular shape in contrast to rectangular projections, because circular regions have mostly closed, high-intensity borders. On the other hand, rectangular projections have diffused borders with many cases where segmentation is not possible, especially where open borders are present. In terms of the type of algorithm, it was observed that unsupervised algorithms are superior to the rest. This project was programmed with no special hardware in MATLAB R2015b on a MacBook Pro (OSX version 10.14) with 16 GB ram and a 2.2 GHz Intel Core i7. With this standard equipment, our solution requires, on average, 3.3 s per particle for segmentation and identification. This can be significantly improved with solutions implemented in C or C++. More information of our implementation can be found in the Supplementary Section.

## 5. Conclusions

We presented an unsupervised segmentation and particle picking algorithm based on matrix decomposition (NNMF) properties. One main advantage was the processing speed because the structure data were not necessary, contrary to the standard methods which need very detailed prior information on shape. Our proposal seeks to find every particle with closed, valid borders; however, two parameters are needed: (1) segmented particle size and (2) radius search. Both parameters may be self-tuned, but we did not consider this possibility because of the training processes required. There were two main results: (1) particle centroids and (2) orientation of the borders. Standard algorithms only provide centroids, and orientation is the result of morphological and chromatic analysis through cepstrum decomposition. The latter technique allows for closed or semi-closed regions to be determined with a high likelihood of included particles. We used 30 images from a keyhole limpet hemocyanin dataset of 680 positive classified regions; the true positive rate was 95.1% for top views and 77.8% for side views. The false negative rate was 14.3%. Although the false positive rate was high at 21.8%, it can be lowered with a supervised classification technique. Furthermore, segmentation obtained 82.2% of true positive classified particles at a 4 pixel distance from real centroids. This last result can be improved with a template-matching technique starting from our solution.

## Figures and Tables

**Table 1:** Performance of the keyhole limpet hemocyanin (KLH) dataset with multiple approaches, top view.

Authors	Reference	FPR%	TPR%	Approach
Zhu et al. (2004)	[43]	13.7	90.3	Unsupervised
Yu and Bajaj (2004)	[45]	24.7	91.7	Unsupervised
Sorzano et al. (2009)	[12]	9.3	69.1	Supervised (Ensemble)
Abrishami et al. (2013)	[46]	16.2	93.3	Supervised (SVM)
Scheres (2015)	[3]	12	90	Semi-supervised
Zhu et al. (2017)	[20]	10	90	Supervised (Deep Learning)
Proposed (2019)		14.3	95.1	Unsupervised

## Supplementary Materials

The following are available online at https://www.mdpi.com/2218-273X/9/12/809/s1; Our code is available online at www.vizzion.cl/projects. Video supplementary is the segmentation.
